# In-Silico assessment of aortic valve function and mechanics under hypertension

**DOI:** 10.3389/fcvm.2025.1692583

**Published:** 2025-11-14

**Authors:** Jason A. Shar, Philippe Sucosky

**Affiliations:** 1 Rand Simulation, Charlottesville, VA, United States; 2 Department of Mechanical Engineering, Kennesaw State University, Marietta, GA, United States

**Keywords:** hypertension, aortic valve, hemodynamics, calcific aortic valve disease, fluid-Structure interaction

## Abstract

**Introduction:**

Calcific aortic valve disease (CAVD) is the most common heart valve disorder. While hypertension is a major risk factor, the mechanisms by which elevated blood pressure contributes to calcification are largely unknown. Given the established sensitivity of aortic valve (AV) tissue to mechanical cues, hypertension may subject AV leaflets to a stress state conducive to CAVD. To address this hypothesis, the objective of this study was to compare AV function and mechanics under normotensive (NTN, 120/80 mmHg), pre-hypertensive (preHTN, 125/80 mmHg) and hypertensive (HTN, 130/90 mmHg) conditions using fluid-structure interaction modeling.

**Methods:**

AV flow and leaflet dynamics were computed in an idealized aortic root geometry using the arbitrary Lagrangian-Eulerian approach. Boundary conditions achieving physiologic cardiac output and coronary perfusion, proper leaflet coaptation, and reflecting preHTN and HTN aortic pressure elevations were determined and applied. The fluid wall shear stress (fWSS) on the leaflet fibrosa was analyzed in terms of regional temporal shear magnitude (TSM) and oscillatory shear index (OSI). Leaflet mechanics was characterized in terms of leaflet profile, coaptation angle, and regional tensile stretch (tS) ratios.

**Results:**

Hypertensive conditions increased early diastolic flow vorticity and decreased the leaflet coaptation angle in a pressure-dependent manner. PreHTN and HTN subjected all leaflets to fWSS overloads (up to 45% increase in radial TSM vs. NTN). While preHTN and HTN resulted in contrasted radial TSM alterations in the leaflet base (up to 0.6- and 1.3-fold change, respectively, vs. NTN), both conditions caused an increase in radial TSM in the belly and tip regions (up to 1.5-fold increase vs. NTN). Radial fWSS bidirectionality increased in a pressure-dependent manner in the base of the left- and non-coronary leaflets (up to 0.23-point increase in OSI vs. NTN) but was attenuated in the belly region (up to 0.19-point decrease). Hypertension caused a pressure-dependent increase in tS ratio (up to 5% increase vs. NTN) on the left- and non-coronary leaflets.

**Discussion:**

Hypertension subjects AV leaflets to complex fluid and structural stress alterations. The results support the existence of a mechano-etiology for CAVD in hypertensive patients and could explain the prevalence of this disease in this patient population.

## 1 Introduction

Calcific aortic valve disease (CAVD) is the most common aortic valve (AV) disease, and ranges from mild leaflet thickening to severe leaflet calcification (1). It is prevalent in 0.4% of the general population and 1.7% of the geriatric population (2). When left untreated, the disease causes obstruction to valvular blood flow and reduces valvular functionality, leading to several complications such as stroke, heart attack, aortic aneurysm, left ventricular hypertrophy and ultimately death (3). The pathobiology of CAVD is driven by a dysfunction of the leaflet endothelial cells (VECs) and the perpetuation of a pro-inflammatory state promoting the switch of the valve interstitial cells (VICs) from a quiescent fibroblast phenotype to activated myofibroblast- and osteoblast-like phenotypes, resulting ultimately in fibro-calcific leaflet remodeling (4).

With a prevalence of 30%–40% in young patients with aortic stenosis (5) and 75% and higher in older patients (6, 7), hypertension is recognized as a major risk factor for CAVD (8–11). Normal arterial blood pressure in adults varies between 120 mmHg during systole and 80 mmHg during diastole. Hypertension is defined as a persistent increase in systemic blood pressure and is graded in three stages (12): pre-hypertension (120–129/<80 mmHg), stage-1 hypertension (130–139/80–89 mmHg) and stage-2 hypertension (>140/>90 mmHg). While the association between hypertensive states and CAVD has been clearly demonstrated, the specific mechanisms contributing to CAVD development in hypertensive patients remain largely unknown (4, 13). AV leaflets experience a rich and complex set of mechanical cues including fluid wall shear stress (fWSS) and structural tensile stretch (tS) that are temporally-, spatially- and directionally-dependent. As shown *in vitro*, VECs and VICs are sensitive to their mechanical environment and respond to mechanical changes by switching their phenotype and altering their biosynthetic activity (14). The leaflet fibrosa is more sensitive to changes in fWSS magnitude, directionality and frequency than the ventricularis and responds to those via cell-adhesion molecule upregulation, and bone-morphogenic protein- and transforming growth factor-dependent increases in pro-inflammatory cytokine production and collagenase activity (15–19). AV leaflets are also sensitive to tS magnitude and frequency, with supra-physiologic stretch contributing to extracellular matrix (ECM) degeneration via alterations in proteolytic enzyme expression and activity (20, 21), osteogenesis via bone-morphogenic protein signaling (22), and VIC phenotypic switch from a contractile/myofibroblast phenotype to a more synthetic phenotype (23).

Hypertensive states subject AV leaflets to a supra-physiologic pressure load during diastole (24), which may result in the same tS and fWSS alterations as those contributing to the progressive loss of valvular homeostasis, as reported above (8, 25). In fact, an *in vitro* study investigated the dynamic deformation characteristics of porcine AV leaflets subjected to hypertensive (120/160 mmHg) and severe hypertensive (150/190 mmHg) conditions using a dual-camera photogrammetry method, and demonstrated up to 10% increase in diastolic radial and circumferential stretch relative to normotensive (80/120 mmHg) conditions (24). However, the demonstration of a potential mechano-etiology of CAVD in hypertensive patients requires the detailed investigation of the hemodynamic and structural alterations caused by elevated blood pressure on AV leaflets, which to date remains limited. Computational fluid-structure interaction (FSI) modeling is uniquely suited to elucidate AV leaflet dynamics under various flow conditions, while providing excellent spatial and temporal resolutions and eliminating subject specificity. Therefore, the objective of this study was to investigate computationally the impact of pre-hypertension (preHTN) and stage-1 hypertension (HTN) on AV hemodynamics and function using an idealized aortic root FSI model including the aortic sinus, the AV leaflets and the coronary circulation.

## 2 Materials and methods

### 2.1 Geometry

**Figure 1:**
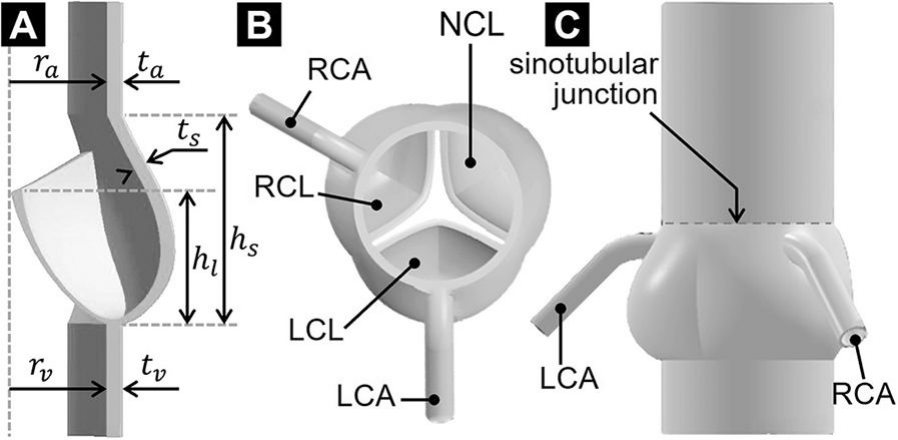
AV geometrical model: **(A)** long-axis cross-sectional view; **(B)** top view; and **(C)** side view.

The aortic root geometry previously designed and published by our group (26) served as the foundation for the model developed in the present study. Briefly, the aortic root included the AV leaflets, the aortic sinuses and the two coronary ostia. The aortic root was reconstructed parametrically using published human dimensions for key anatomical features (left-ventricular outflow tract and aortic diameters, aortic wall thickness, sinus height and diameter, leaflet height, Table 1) (27, 28). The AV leaflet geometry was generated by considering different anatomical landmarks (e.g., tip point, centerline, commissure point, annulus points) and the native leaflet regional thickness (29) (Figure 1A). Three identical leaflets were created using this approach and placed within the aortic root geometry to model the left-coronary leaflet (LCL), right-coronary leaflet (RCL) and non-coronary leaflet (NCL) in a nearly closed position (Figure 1B). Coronary ostia were placed 18 mm above the aortic annulus (30), and the left- and right-coronary arteries (LCA and RCA, respectively) were modeled as circular conduits [LCA diameter: 4.0 mm; RCA diameter: 3.2 mm (30, 31)] extending 20 mm away from the aortic wall. The aortic outlet was also modeled as a circular conduit extending 36 mm from the sinotubular junction to improve flow development and numerical stability (Figure 1C).

**Table 1:** Aortic root and leaflet thickness characteristics.

Aortic root dimensional characteristics [mm]							Leaflet thickness characteristics [mm]				
*r_a_*	*r_v_*	*h_s_*	*h_l_*	*t_a_*	*t_s_*	*t_v_*	Attachment edge	Belly	Coaptation zone	Free edge	Nodulus of Aranti
12.5	12.5	22.1	14.0	1.5	1.5	1.5	1.16	0.18–0.58	0.68–1.29	1.53	2.06

### 2.2 Constitutive models

The leaflet material was modeled as isotropic and homogenous. The non-linear hyperelastic strain-stress leaflet response was approximated using a three-parameter Mooney-Rivlin model with strain energy density function defined as

$$W=C_{10}({\bar{I}}_{1}-3)+C_{01}({\bar{I}}_{2}-3)+C_{11}({\bar{I}}_{1}-3)({\bar{I}}_{2}-3),$$

where ${\bar{I}}_{1}$ and ${\bar{I}}_{2}$ are the two invariants of the Cauchy-Green deformation tensor, and *C*_10_ = 32,823 Pa, *C*_01_ = 2,955.1 Pa and *C*_11_ = 585,790 Pa are material constants calibrated against published mechanical test data (32). The aortic wall was modeled as a nearly incompressible isotropic and linear elastic material (density: 1,080 kg/m^3^; Young's modulus: 2 MPa; Poisson's ratio: 0.45) (33). Consistent with previous valvular and vascular flow models (34–36), a fluid density of 1,050 kg/m^3^ and dynamic viscosity of 0.0035 Pa·s were adopted to approximate blood as a Newtonian, homogenous and incompressible fluid.

### 2.3 Boundary conditions

Three sets of boundary conditions were developed to simulate normotensive (NTN, 120/80 mmHg), pre-hypertensive (preHTN, 125/80 mmHg), and stage-1 hypertensive (HTN, 130/90 mmHg) conditions.

#### 2.3.1 Normotensive conditions

**Figure 2:**
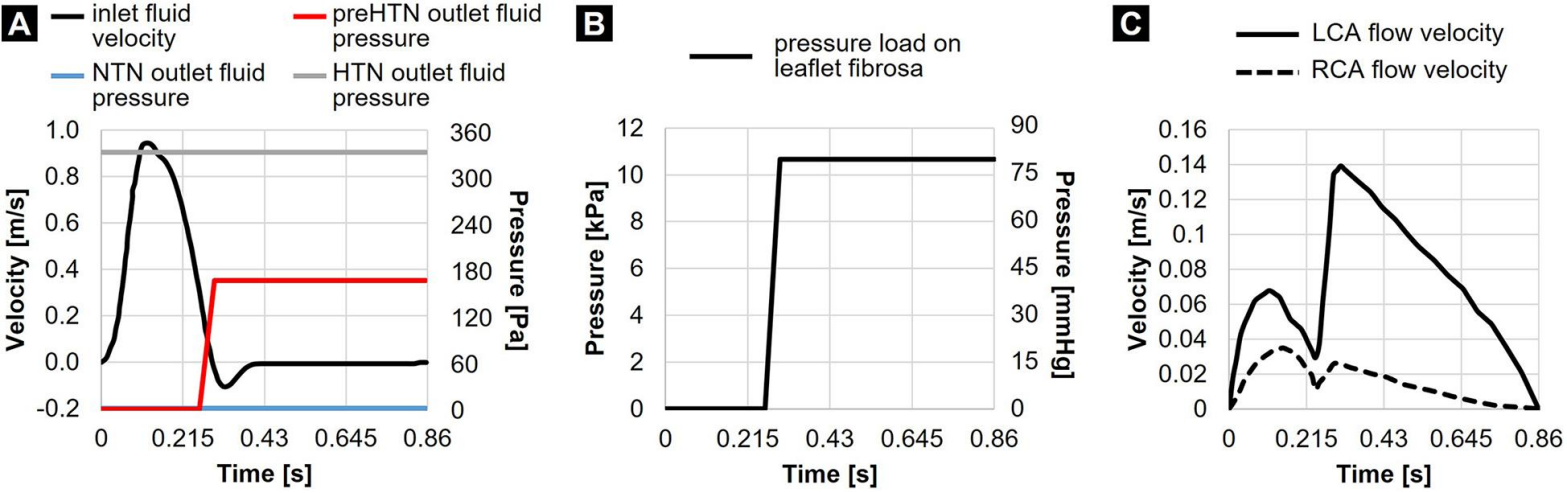
Flow and structural boundary conditions: **(A)** inlet fluid velocity and outlet fluid pressures prescribed in the NTN, preHTN, and HTN models; **(B)** pressure load prescribed on the leaflet fibrosa in all models; and **(C)** coronary flow velocities.

Physiologic flow was achieved by prescribing zero gage pressure at the outlet of the fluid domain and a transient and spatially uniform velocity profile at the inlet, resulting in a cardiac output of 5 L/min, a cardiac cycle of 0.86 s and a systolic-to-diastolic ratio of 1:2 (Figure 2A). While this modeling strategy effectively replicated physiologic systolic flow and leaflet opening under a near-zero transvalvular pressure gradient, it prevented the generation of a physiologic negative transvalvular pressure during diastole leading to leaflet closure and coaptation. This was compensated for by subjecting the aortic surface of the leaflets to a numerical coaptation constraint consisting of a uniform pressure load of 80 mmHg (10.7 kPa) during diastole (Figure 2B). Coronary flow velocities calculated from published physiologic coronary flow rates (LCA: 0.2 L/min and RCA: 0.05 L/min) (37) were specified at the coronary outlets (Figure 2C). The same LCA and RCA flow rates were considered in all three models to simulate the autoregulatory capacity of the coronary circulation, which results in the maintenance of a relatively constant myocardial perfusion across a wide range of coronary perfusion pressure (38). The leaflet surfaces were defined as fluid-structure interfaces. To preserve a single continuous fluid domain throughout the cardiac cycle while maintaining numerical stability, we resolved the structural contact between the leaflets by implementing a frictionless contact with a contact offset of 0.5 mm in ANSYS Mechanical.

#### 2.3.2 Pre-hypertensive and hypertensive conditions

While the NTN model operated with a zero-gauge pressure at the aortic fluid outlet, different pressure conditions had to be determined to replicate the increase in aortic blood pressure in the preHTN and HTN cases. The determination of the appropriate fluid outlet conditions (${\tilde{P}}_{\mathrm{aortic}}$) resulting in an effective increase in aortic pressure ($P_{\mathrm{aortic}}$) of +5 mmHg and +10 mmHg under preHTN and HTN, respectively, required: 1) the assessment of the actual time-averaged pressure drop across the NTN valve model (${\bar{\Delta P}}_{\mathrm{NTN}}=-11\;\;\mathrm{mmHg}$), 2) its comparison to the physiologic time-averaged pressure drop across the native AV [${\bar{\Delta P}}_{\mathrm{physio}}=-46\;\;\mathrm{mmHg}$ as calculated on a standard Wiggers diagram (39)] via the calculation of a pressure scaling factor ($\beta ={\bar{\Delta P}}_{\mathrm{NTN}}/{\bar{\Delta P}}_{\mathrm{physio}}=0.24$), and 3) the determination of a scaled gauge pressure to be prescribed at the aortic outlet (${\tilde{P}}_{\mathrm{aortic}}=\beta \times P_{\mathrm{aortic}}$). This strategy resulted in an aortic gauge pressure of 1.2 mmHg (160 Pa) and 2.4 mmHg (320 Pa) in the preHTN and HTN case, respectively. Since the increase in aortic pressure under preHTN is only effective during systole, this boundary condition was only enforced during systole. In contrast, for HTN, which imposes a sustained elevated aortic pressure, this fluid pressure load was maintained throughout the cardiac cycle (Figure 2A).

### 2.4 Mesh generation

The structural and fluid domains were spatially discretized using tetrahedral elements. The structural grid consisted of 48,516 tetrahedral elements (element size: 0.57 mm), which was determined from a previous mesh sensitivity analysis performed on a similar valve geometry (40, 41). For the fluid domain, a mesh sensitivity analysis was performed to determine the appropriate element size. Briefly, the fluid domain was first discretized using an element size of 1.0 mm. FSI simulations were run until peak systole. The geometry of the fluid domain was captured at that time with the valve in its fully open state, and meshed using four different element sizes (1.0, 0.75, 0.64 and 0.55 mm). Steady-state computational fluid dynamics (CFD) simulations representative of peak-systolic conditions (flow rate: 25 L/min) were then performed to quantify the effect of mesh element size on the flow velocity at a point located 21 mm downstream of the inlet, the pressure drop across the model, and the surface-averaged pressure on the NCL. The results indicated that the smallest element size generated less than 1% change in each parameter as compared to the previous grid size (Table 2). Therefore, an element size of 0.64 mm, which resulted in a computational grid of 629,284 elements, was adopted to mesh the fluid domain of all FSI models. While the mesh sensitivity analysis was performed at peak systole, its validity is expected to extend throughout the cardiac cycle due to the dynamic remeshing method that was selected to maintain the initial mesh size distribution during mesh motion. By controlling local refinement, limiting skewness and preserving original element sizing, this remeshing method ensured the maintenance of a consistent mesh quality throughout the cardiac cycle even as elements are added or removed.

**Table 2:** Fluid mesh sensitivity analysis.

Element size (mm)	Grid size (elements)	Average velocity at a point (m/s)	Average NCL pressure (Pa)	Transvalvular pressure drop (Pa)
1.00	162,604	0.73	21.09	173.84
0.75	452,000	0.69	31.46	169.23
0.64	629,284	0.67	27.17	164.78
0.55	926,672	0.67	27.19	163.80

### 2.5 FSI strategy

The momentum transfer between blood flow and the leaflets was achieved via a two-way FSI modeling strategy implementing the arbitrary Lagrangian-Eulerian (ALE) method (36). Briefly, this numerical approach consists of altering the topology of the fluid mesh by fitting it with the moving structure (42). The governing equations for the fluid (Navier–Stokes and continuity equations) were solved in ANSYS Fluent (Canonsburg, PA, USA) using the finite volume method and the pressure-based coupled solver. The flow was modeled as laminar. The spatial and temporal discretization of the governing equations was obtained by the second order upwind scheme. The solver iteration ended once the scaled convergence residual decreased to 10^−3^ and 10^−5^ for the momentum and continuity equations, respectively. The deformation of the structural domain was governed by the momentum equation. The displacements were solved in ANSYS Mechanical, which uses a direct-displacement finite element method and the Newton-Raphson method to iteratively solve the equilibrium equations until convergence is achieved. Continuity of displacement, velocity, and traction at the fluid-structure interface were enforced via three coupling conditions in ANSYS System Coupling. Structural-fluid coupling was considered converged when the difference between two successive data transfers fell below 10^−3^ for both displacement and force. Due to the dynamic nature of the fluid mesh, the degradation of cells due to the large displacement of the leaflet boundary was compensated for by enabling mesh smoothing and remeshing. The time step size was manually adjusted over the cardiac cycle to balance stability, accuracy, and computational cost. During systole, smaller time steps were required to resolve the sharp velocity gradients associated with peak ejection and to maintain a Courant–Friedrichs–Lewy (CFL) number close to 1. During diastole, when flow fields were slower and often recirculatory, larger steps could be applied without loss of accuracy. This adaptive strategy allowed us to capture transient flow and structural responses while maintaining numerical stability. The simulation end time was 1.72 s to span two cardiac cycles. Results were extracted at 86 time steps during the second period (equivalent time step of 10 ms).

### 2.6 Valvular function characterization

The validity of the normotensive model to simulate native AV dynamics was assessed by comparing the predicted geometric orifice area (GOA), effective orifice area (EOA), peak systolic jet velocity and mean pressure gradient against published values. The GOA, which characterizes the anatomical orifice area of the valve at peak systole, was computed by measuring the area of the planar region connecting the lowest points of the free edge of the leaflets. The EOA, which defines the cross-sectional area of the jet downstream of the orifice at peak systole, was calculated as:

$$\mathrm{EOA}=\frac{Q}{51.6\sqrt{\Delta P}},$$

where *Q* is volume flow rate in mL/s, and ΔP is the transvalvular pressure drop in mmHg.

### 2.7 Valvular hemodynamics characterization

The specific characteristics of AV vorticity dynamics have been shown to contribute to valvular function and the rapid closure of the leaflets during diastole. Therefore, AV hemodynamics was characterized using in-plane vorticity and velocity vector fields captured in the three vertical planes bisecting the leaflets. The fWSS on the leaflet fibrosa was characterized in terms of temporal shear magnitude (TSM) and oscillatory shear index (OSI) defined as:

$$\mathrm{TSM}=\frac{1}{T}\int_{0}^{T}|\tau |\;dt$$

and

$$\mathrm{OSI}=\frac{1}{2}\left[1-\frac{\left|\int_{0}^{T}\tau \;dt\right|}{\int_{0}^{T}|\tau |\;dt}\right],$$

respectively, where τ is the instantaneous fWSS magnitude, and *T* is the cardiac period. Those metrics were computed in the tip, belly, and base regions of each leaflet for both the radial and circumferential fWSS components.

### 2.8 Valvular mechanics characterization

The effects of hypertension on leaflet kinematics was investigated qualitatively by comparing the profile of each leaflet captured at discrete time points between all three models. The coaptation configuration of each leaflet was characterized in terms of the coaptation angle $\theta_{c}$ defined as the angle between the plane containing the valve annulus and the line tangent to the leaflet base in the plane bisecting the leaflet of interest. Leaflet deformation characteristics in the radial and circumferential directions were assessed regionally in terms of the leaflet tS ratio (λ), which represents the ratio of the deformed length (l) to the initial length (L) of a line element aligned along a particular direction:

$$\lambda =\frac{l}{L}$$

## 3 Results

### 3.1 NTN model validation parameters

The NTN model generated a peak-systolic EOA of 3.84 cm^2^ and a mean systolic pressure gradient of 3.46 mmHg. At peak systole (*t* = 0.13 s), the maximum jet velocity and GOA were 1.15 m/s and 4.47 cm^2^, respectively. The deceleration phase (*t* = 0.27 s), was associated with a 36% reduction in orifice jet velocity (0.74 m/s) and a 24% decrease in GOA (3.40 cm^2^) relative to peak-systole.

### 3.2 Flow vorticity and velocity fields

**Figure 3:**
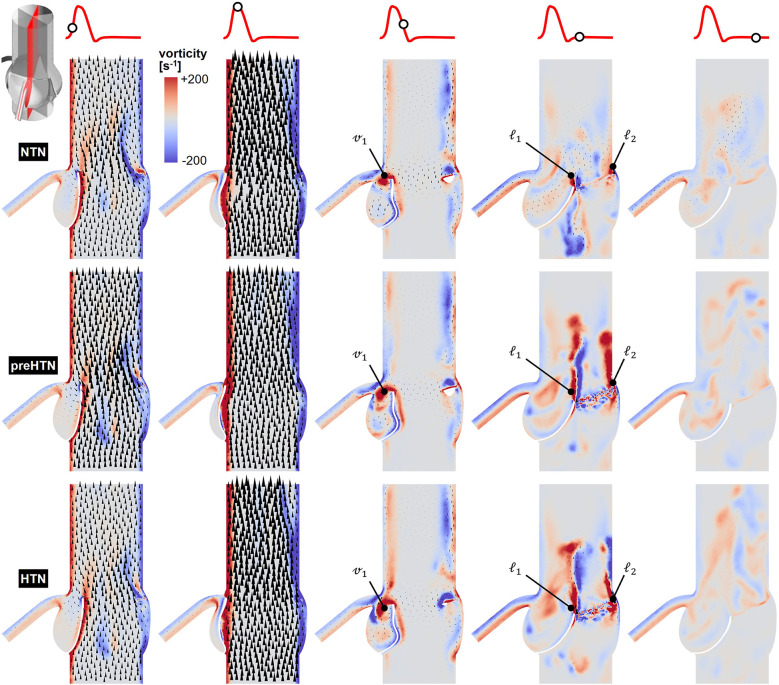
Snapshots of the velocity vector and vorticity contour fields captured in the plane bisecting the LCL in the NTN, preHTN and HTN models during the acceleration phase (*t* = 0.08 s), peak systole (*t* = 0.13 s), the deceleration phase (*t* = 0.27), early diastole (*t* = 0.40 s), and late diastole (*t* = 0.56 s).

**Figure 4:**
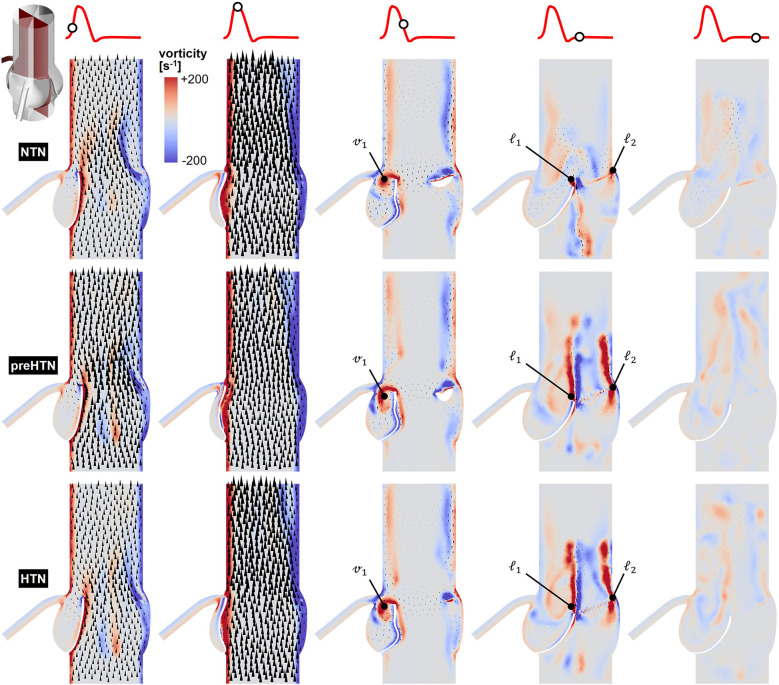
Snapshots of the velocity vector and vorticity contour fields captured in the plane bisecting the RCL in the NTN, preHTN and HTN models during the acceleration phase (*t* = 0.08 s), peak systole (*t* = 0.13 s), the deceleration phase (*t* = 0.27), early diastole (*t* = 0.40 s), and late diastole (*t* = 0.56 s).

**Figure 5:**
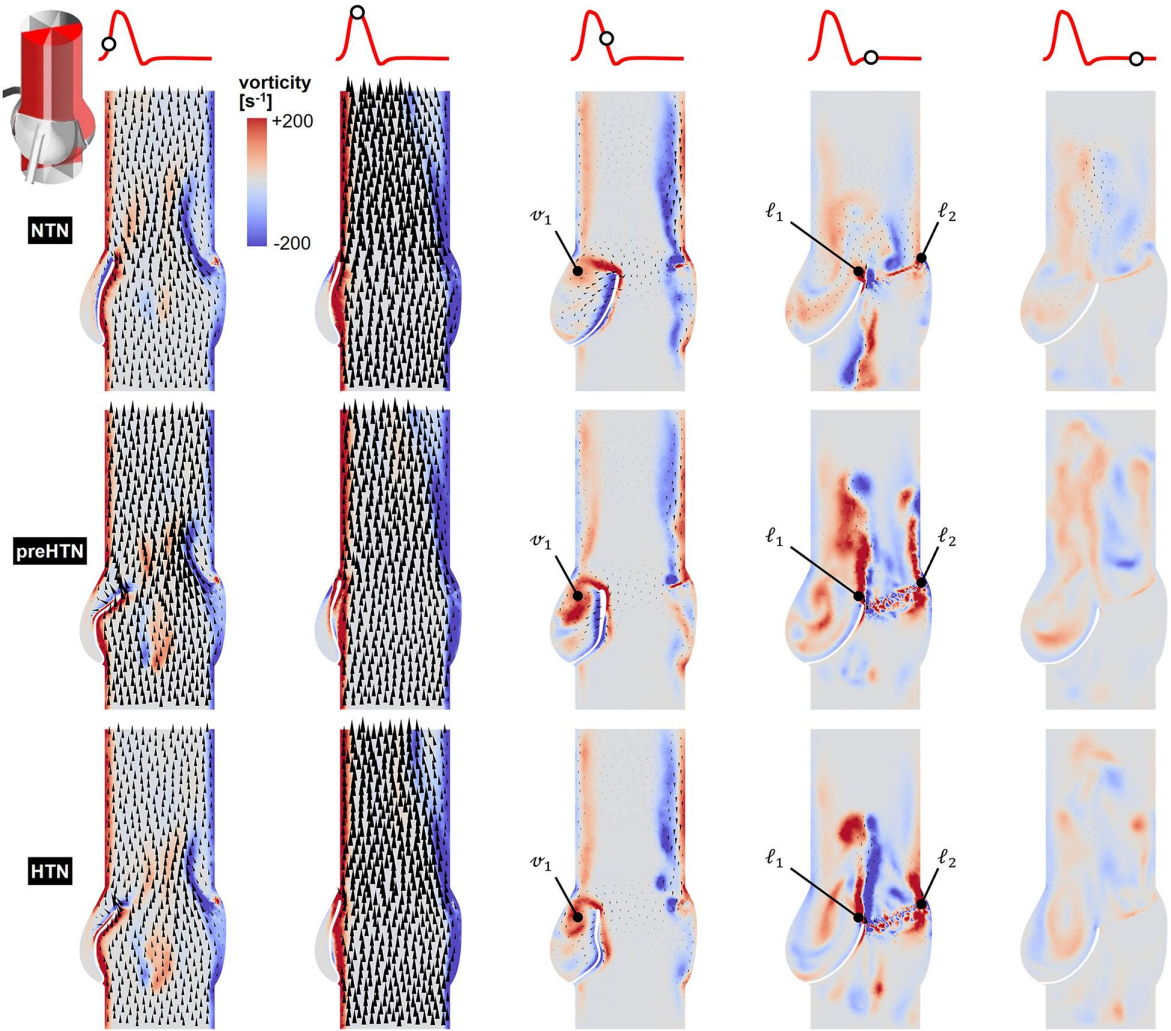
Snapshots of the velocity vector and vorticity contour fields captured in the plane bisecting the NCL in the NTN, preHTN and HTN models during the acceleration phase (*t* = 0.08 s), peak systole (*t* = 0.13 s), the deceleration phase (*t* = 0.27), early diastole (*t* = 0.40 s), and late diastole (*t* = 0.56 s).

Snapshots of the in-plane vorticity contour and velocity vector fields captured during the acceleration phase (*t* = 0.08 s), at peak systole (*t* = 0.13 s), during the deceleration phase (*t* = 0.27 s), early diastole (*t* = 0.40 s), and late diastole (*t* = 0.56 s) in the vertical planes bisecting the LCL, RCL and NCL are shown in Figures 3–5, respectively. Animations of the vorticity and velocity fields during one cardiac cycle are available in Supplementary Video S1.

Systolic hemodynamics was weakly impacted by the pressure condition (<8% difference in in-plane vorticity between NTN, preHTN and HTN). All models resulted in similar flow patterns exhibiting jet-like characteristics including the existence of a unidirectional and streamlined flow, and the presence of shear layers extending from the leaflet tips. As expected, vorticity was mainly concentrated along the aortic and leaflet walls as well as in the shear layers, where velocity gradients are particularly high. During the deceleration phase, the flow was also relatively consistent across all models but exhibited more complex flow features. The interactions between the valvular jet, the near-stagnant sinus flow and the moving leaflets generated vortices near the leaflet tips (feature ℓ_1_), which rolled up and migrated toward the sinus wall. The size and penetration distance of those vortices into the sinuses increased in a pressure-dependent manner.

Early diastolic hemodynamics exhibited the strongest dependence on aortic pressure. Shortly after leaflet coaptation, the rapid downward deflection of the leaflets caused by the hammer effect and the sudden backflow of blood from the aorta gave rise to shear layers in the wake of the leaflet tips (feature $\ell_{1}$) and in the vicinity of the commissure regions (feature $\ell_{2}$). While those structures were relatively small and localized near the aortic side of each leaflet in the NTN model, they were larger and penetrated further into the downstream aortic flow in the preHTN and HTN models (increase in in-plane vorticity: +126% vs. NTN). Late diastolic flow patterns were similar across all three models and characterized by the progressive breakdown of the vortical structures and their diffusion into the surrounding flow.

### 3.3 Instantaneous fWSS characteristics

**Figure 6:**
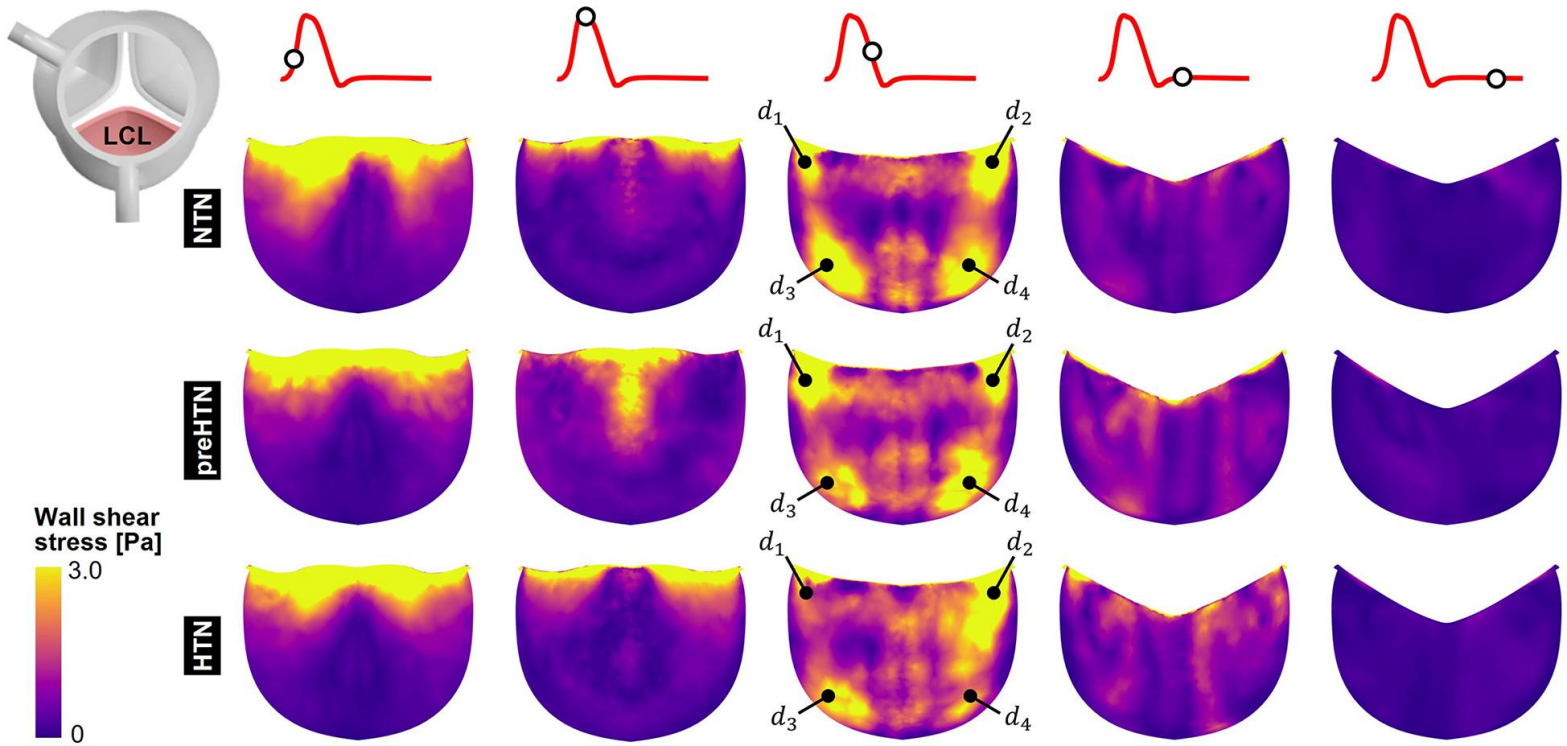
Snapshots of the fluid wall shear stress (fWSS) contours captured on the fibrosa of the LCL in the NTN, preHTN and HTN models during the acceleration phase (*t* = 0.08 s), peak systole (*t* = 0.13 s), the deceleration phase (*t* = 0.27), early diastole (*t* = 0.40 s), and late diastole (*t* = 0.56 s).

**Figure 7:**
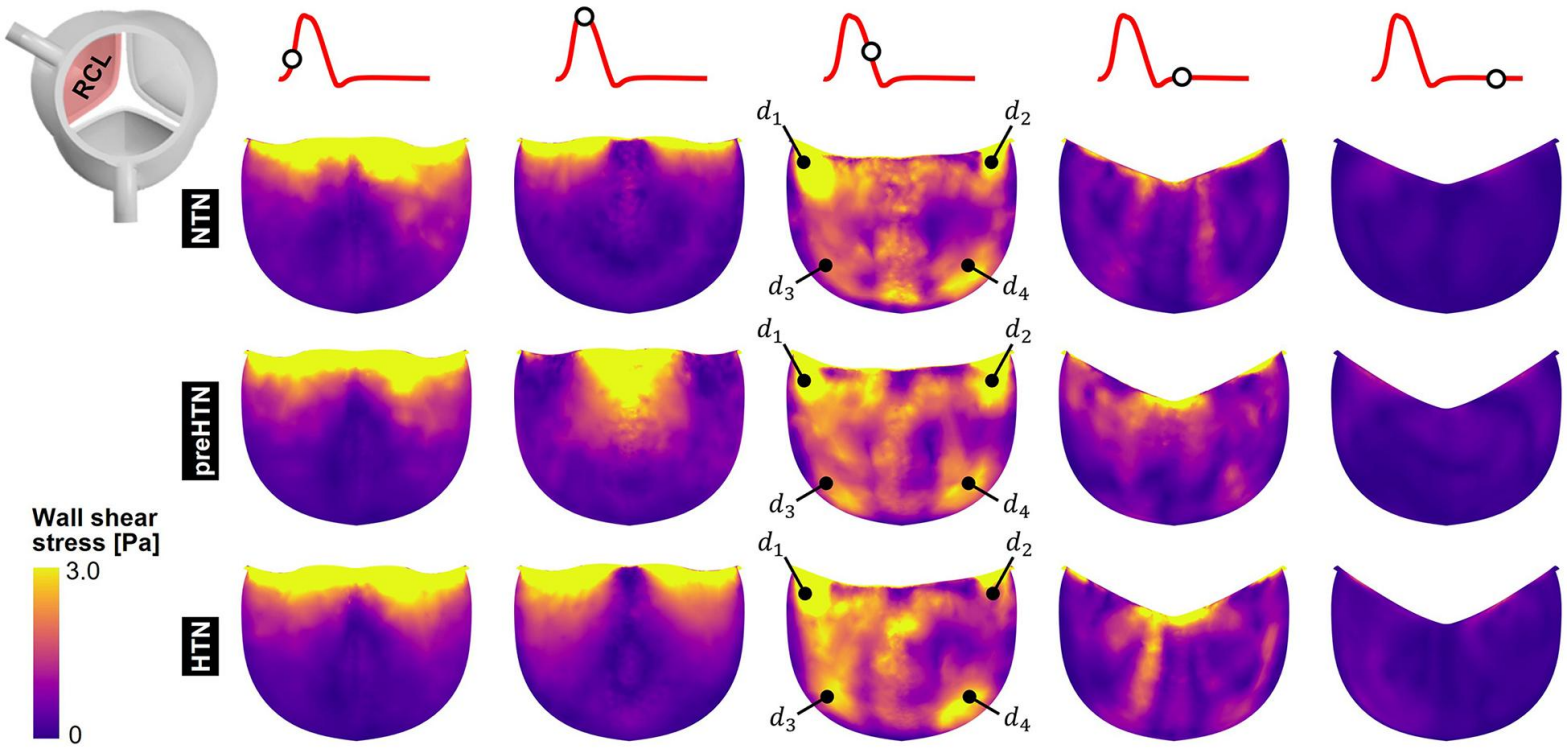
Snapshots of the fluid wall shear stress (fWSS) contours captured on the fibrosa of the RCL in the NTN, preHTN and HTN models during the acceleration phase (*t* = 0.08 s), peak systole (*t* = 0.13 s), the deceleration phase (*t* = 0.27), early diastole (*t* = 0.40 s), and late diastole (*t* = 0.56 s).

**Figure 8:**
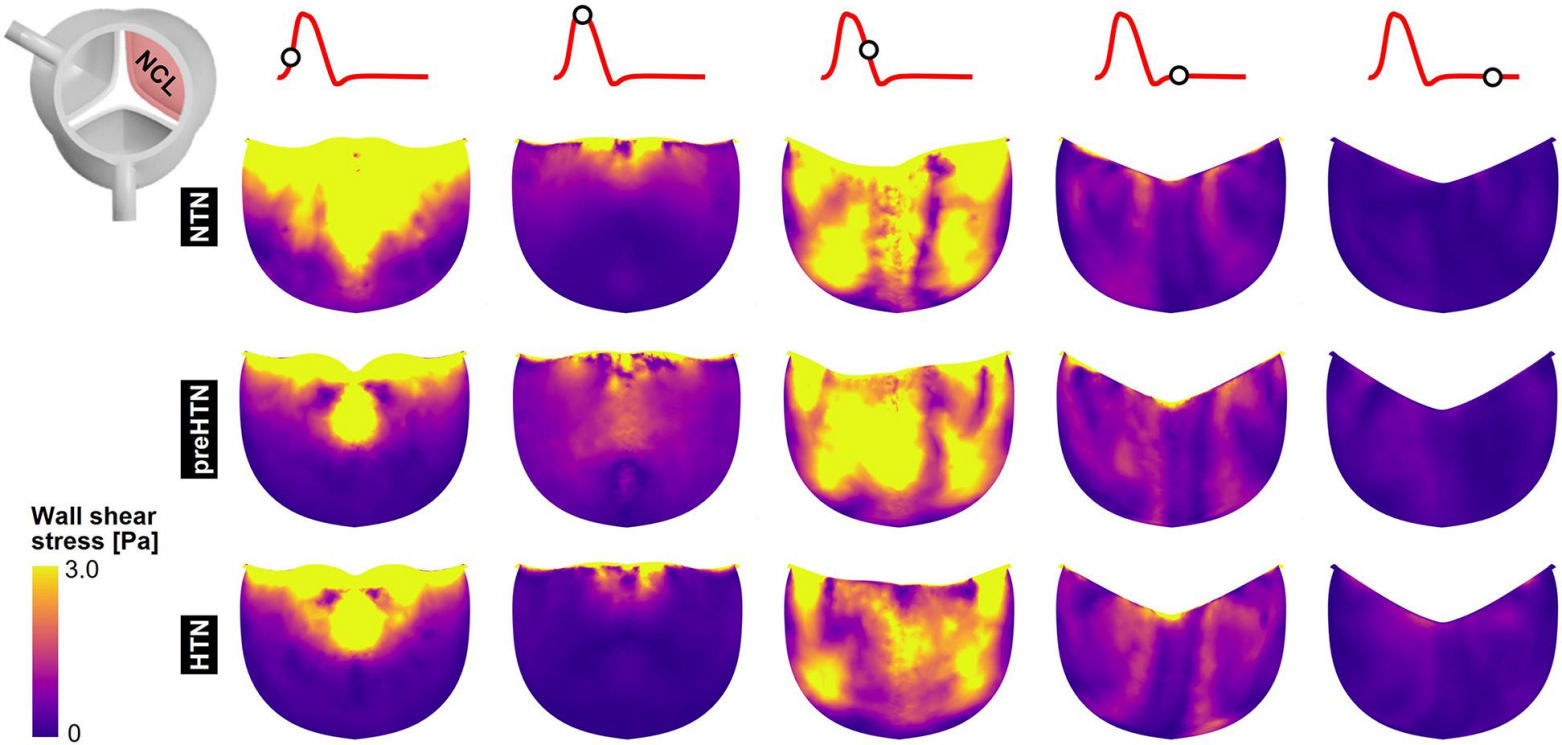
Snapshots of the fluid wall shear stress (fWSS) contours captured on the fibrosa of the NCL in the NTN, preHTN and HTN models during the acceleration phase (*t* = 0.08 s), peak systole (*t* = 0.13 s), the deceleration phase (*t* = 0.27), early diastole (*t* = 0.40 s), and late diastole (*t* = 0.56 s).

Snapshots of the fWSS contours captured on the fibrosa of the LCL, RCL and NCL throughout the cardiac cycle are shown in Figures 6–8, respectively. Animations of the fWSS contour fields are available in Supplementary Video S2. Inspection of the contour fields indicated the strong dependence of the fWSS spatial distribution on both the pressure condition and the leaflet. Regardless of the pressure condition, the coronary leaflets (LCL and RCL) exhibited relatively similar fWSS fields. During the acceleration phase, high fWSS levels were concentrated in the coaptation region near the tip of the LCL and RCL and remained low in the base and belly regions, in all three models. In contrast, the NCL exhibited more widespread elevated fWSS levels in the tip and belly regions in the NTN model, and hypertension tended to displace those high fWSS regions to the leaflet tip. Peak-systolic conditions were associated with the shrinking of the high fWSS regions and their migration toward the leaflet tip (NTN and HTN models) or leaflet tip and belly regions (preHTN model). During the deceleration phase, high fWSS levels were mostly localized to the four corners of the LCL and RCL (see features $d_{1}$, $d_{2}$, $d_{3}$, $d_{4}$) and were weakly impacted by the pressure condition. In contrast, the NCL exhibited widespread high fWSS levels over its entire surface, which were slightly attenuated under HTN conditions. Lastly, early and late diastole were marked by the progressive attenuation of the fWSS on all leaflets, for all pressure conditions.

### 3.4 Time-Averaged fWSS characteristics

**Figure 9:**
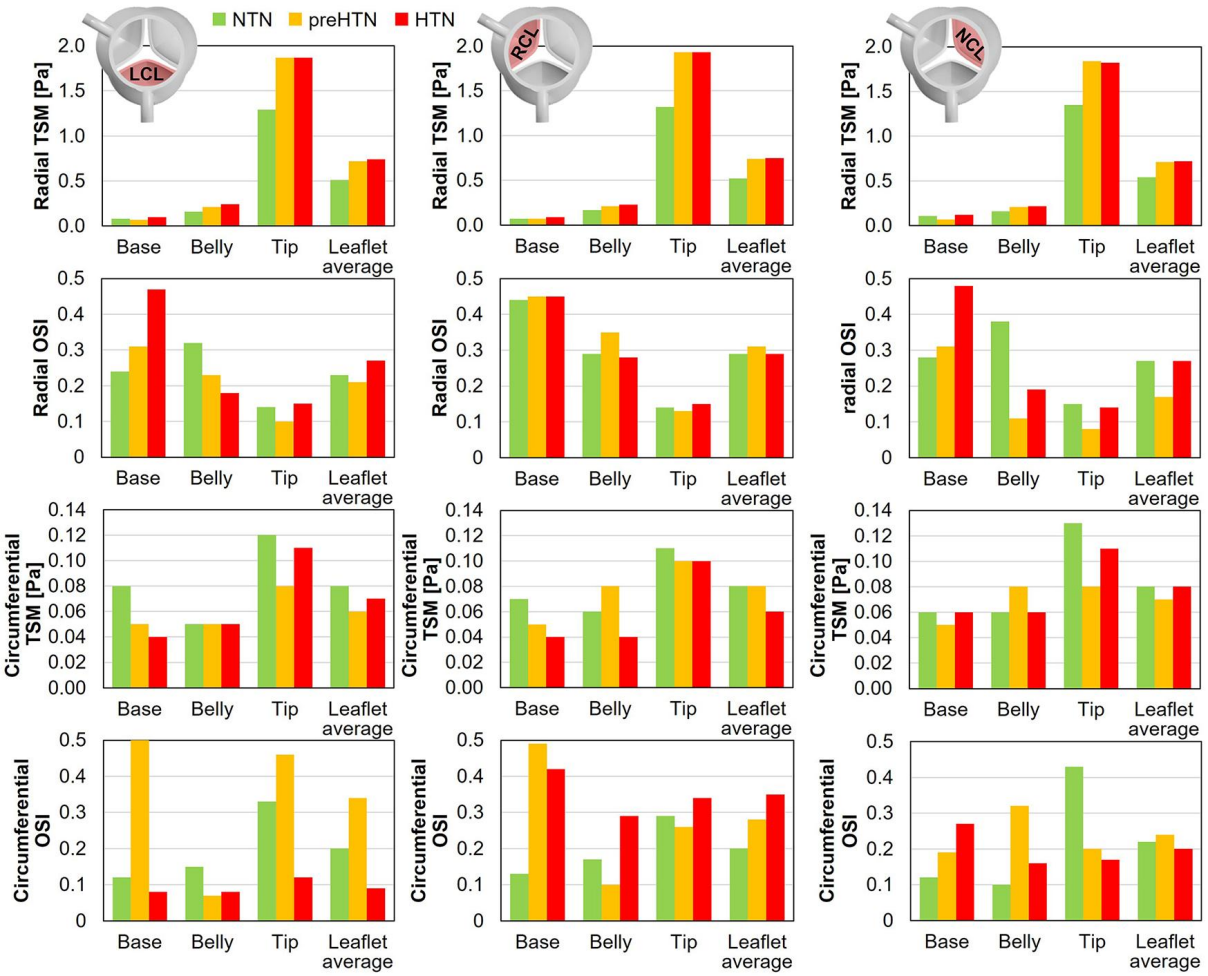
Radial and circumferential temporal shear magnitude (TSM) and oscillatory shear index (OSI) captured in the base, belly and tip of the LCL, RCL and NCL fibrosa in the NTN, preHTN and HTN models.

Time-averaged fWSS characteristics (i.e., TSM and OSI) are shown in Figure 9.

#### 3.4.1 TSM

Inspection of the leaflet-average TSM predictions under normotensive conditions indicates that the fibrosa fWSS was dominated by the radial component (6.1 < radial-to-circumferential TSM ratio < 6.5). Aortic pressure elevation exacerbated this domination (radial-to-circumferential TSM ratio > 9.4 under preHTN and HTN conditions). Overall, elevated pressure conditions subjected all leaflets to radial fWSS overloads. PreHTN caused a 41%, 42% and 31% increase in radial TSM on the LCL, RCL and NCL, respectively, relative to NTN conditions. HTN conditions slightly amplified this phenomenon and resulted in a 45%, 44% and 33% increase, respectively, relative to the NTN model. Aortic pressure had a much weaker effect on the circumferential fWSS component (less than 25% difference in circumferential TSM between NTN and HTN conditions).

The analysis of the regional TSM predictions indicates the strong spatial dependence of the radial fWSS component. Regardless of the leaflet and of the aortic pressure, the minimum radial TSM was systematically predicted in the base of the fibrosa, while the maximum occurred in the tip region (LCL: 16-fold increase in radial TSM from base to tip under NTN conditions; RCL: 19-fold increase; NCL: 12-fold increase). This was not the case for the circumferential component, which was more uniform over the leaflet surface (<2.8-fold increase in circumferential TSM between base and tip regions). Each leaflet region exhibited specific changes in radial fWSS magnitude with aortic pressure elevation. In the base region, preHTN resulted in sub-physiologic radial fWSS magnitudes (0.9-, 1.0- and 0.6-fold change in radial TSM on the LCL, RCL and NCL, respectively, relative to NTN conditions), while HTN imposed supra-physiologic levels (1.3-, 1.3- and 1.1-fold increase, respectively). In the belly region, the increase in radial fWSS magnitude captured in all three leaflets was more substantial than in the base and was pressure magnitude-dependent (preHTN: 1.3-, 1.2- and 1.3-fold change in radial TSM on the LCL, RCL and NCL, respectively, relative to normotensive conditions; HTN: 1.5-, 1.4-, and 1.4-fold increase, respectively). While preHTN and HTN imposed similar radial fWSS overloads in the tip region (up to 1.5-, 1.5- and 1.4-fold change in radial TSM on the LCL, RCL and NCL, respectively, relative to normotensive conditions), those increases were not pressure magnitude-dependent (<1% difference between preHTN and HTN radial TSM).

#### 3.4.2 OSI

OSI predictions under normotensive conditions revealed contrasted characteristics in the radial and circumferential directions. For all leaflets, the radial fWSS was moderately to strongly bidirectional in the base and belly regions (0.24 < OSI < 0.44) and more unidirectional in the tip region (0.14 < OSI < 0.15). This trend was reversed for the non-dominant circumferential fWSS component, which was mostly unidirectional in the base and belly regions (0.10 < OSI < 0.17) and more oscillatory in the tip (0.29 < OSI < 0.43).

PreHTN and HTN conditions resulted in complex component-, region- and leaflet-dependent changes in fWSS directionality. The radial fWSS component captured in the base of the LCL and NCL became progressively more bidirectional under preHTN and HTN (LCL: 0.07- and 0.23-point increase in radial OSI, respectively, vs. NTN; NCL: 0.03- and 0.20-point increase, respectively), while that captured in the belly exhibited increased unidirectionality under HTN (0.14- and 0.19-point decrease in radial OSI on the LCL and NCL, respectively, vs. NTN). Interestingly, the directionality of the radial fWSS captured on the RCL was not impacted by pressure changes. The impact of elevated pressure on the circumferential OSI exhibited even less consistency. The most notable alterations were in the base of the LCL and RCL, where preHTN caused a complete reversal of fWSS directionality from strongly unidirectional to strongly bidirectional (LCL: 0.38-point increase in circumferential OSI vs. NTN; RCL: 0.36-point increase), and in the tip of the LCL and NCL, where HTN attenuated substantially fWSS bidirectionality (LCL: 0.21-point decrease in circumferential OSI vs. NTN; NCL: 0.23-point decrease).

### 3.5 Leaflet kinematics and coaptation

**Figure 10:**
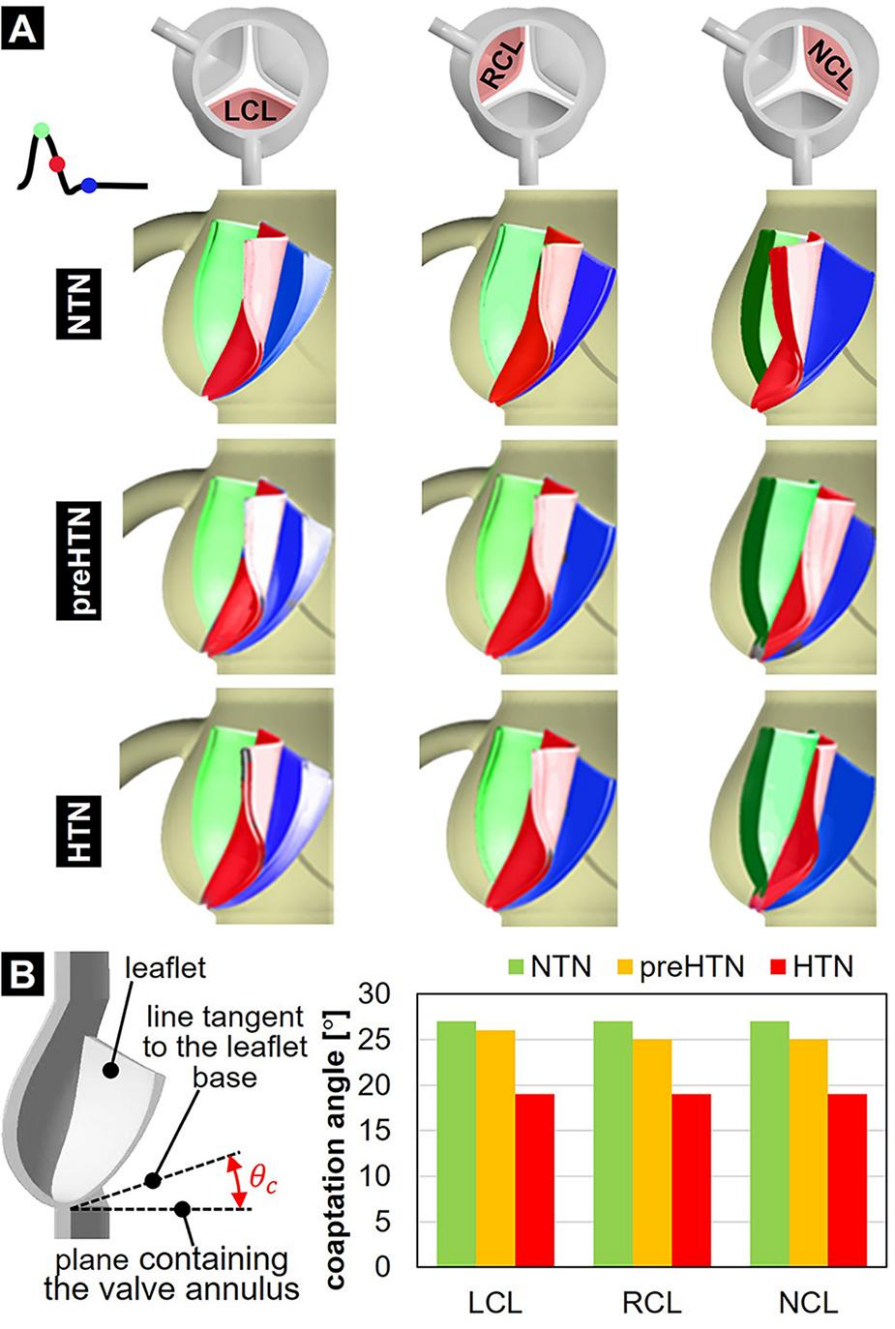
Leaflet deformation characteristics: **(A)** leaflet profiles captured at peak systole (green), during the deceleration phase (red) and during diastole (blue); and **(B)** leaflet coaptation angle predicted in the NTN, preHTN and HTN models.

The impact of aortic pressure on leaflet kinematics is shown in Figure 10A. In the NTN model, all three leaflets shared relatively similar peak-systolic and diastolic profiles and curvatures. During the deceleration phase however, NCL closure lagged behind the LCL and RCL. Elevated pressure conditions did not substantially alter the leaflet curvature and profile at peak systole. In contrast, they caused the NCL to close more rapidly than the coronary leaflets, thereby reversing the trend observed under normotensive conditions. Diastolic leaflet profiles predicted in the preHTN and HTN models were similar but exhibited more curvature than in the NTN model due to the increased fluid pressure acting on the leaflet fibrosa. Those qualitative observations are supported by the quantification of the leaflet coaptation angle (Figure 10B). At any given pressure, coaptation angles were similar across all three leaflets. In contrast, pressure had a strong impact on this metric. The increased leaflet deflection and curvature previously observed during diastole in the preHTN and HTN models translated into a 4% and 30% decrease in leaflet coaptation angle, respectively, relative to the NTN model.

### 3.6 Leaflet deformation characteristics

**Figure 11:**
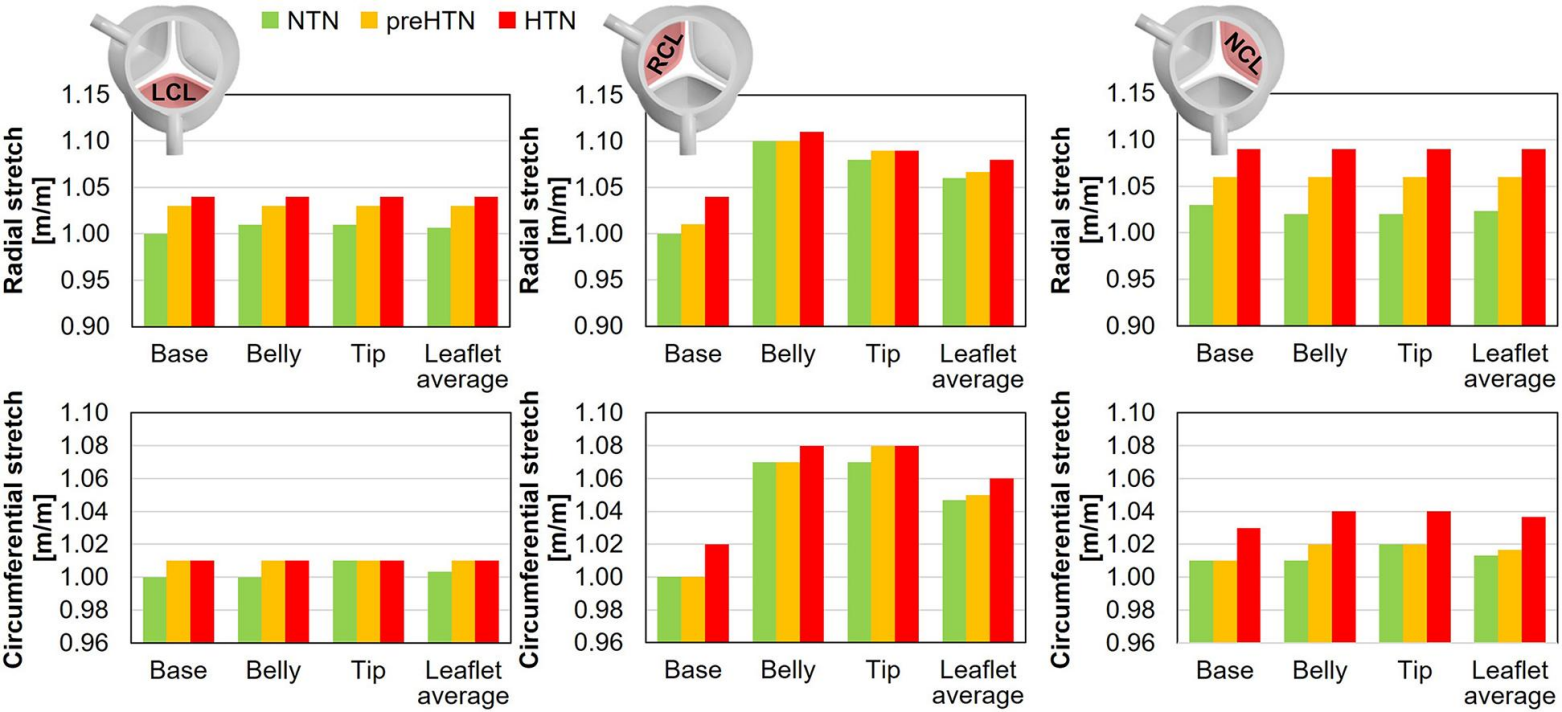
Maximum radial and circumferential stretch captured in the base, belly and tip of the LCL, RCL and NCL fibrosa in the NTN, preHTN and HTN models.

Maximum radial and circumferential tS ratios captured in the coapted leaflets are reported in Figure 11.

Regardless of the aortic pressure, the RCL experienced the largest radial and circumferential deformations during the cardiac cycle (up to 5% difference in tS vs. LCL and NCL). In addition, while the LCL and NCL exhibited relatively spatially-uniform tS distributions (less than 1% variation across the leaflet surface), the RCL was marked by a more spatially dependent stretch field regardless of the pressure condition (up to 10% variation in tS across the leaflet surface). Elevated pressure resulted in a pressure magnitude-dependent increase in leaflet tS, which affected the radial component more substantially than the circumferential component (preHTN: +3% and +1% in radial and circumferential tS, respectively, vs. NTN; HTN: +5% in radial tS and +2% in circumferential tS vs. NTN). The maximum stretch ratios predicted on the RCL were the least impacted by hypertensive conditions (less than 4% and 2% increase in radial and circumferential tS, respectively, vs. NTN). In the base region of each leaflet, which is more prone to calcification, the analysis of the radial and circumferential tS revealed similar characteristics, with supraphysiologic tS levels under preHTN and HTN affecting the radial component more than the circumferential component (LCL: up to 4% increase in radial tS vs. NTN; RCL: up to 4% increase; NCL: up to 6% increase).

## 4 Discussion

In this study, a two-way FSI modeling strategy to simulate the effects of pre-hypertension and stage-1 hypertension on aortic valve function and dynamics. This section discusses the validity of the computational model, the effects of hypertension on valvular hemodynamics, and the potential clinical implications of hypertension on valvular pathogenesis.

### 4.1 Model validity

Key hemodynamic parameters predicted in the NTN model, such as the mean pressure gradient (3.46 mmHg), peak-systolic jet velocity (1.15 m/s), EOA (3.84 cm^2^) and GOA (4.47 cm^2^), were consistent with clinical and experimental data (mean pressure gradient: <5 mmHg, peak systolic jet velocity: <2 m/s, EOA: 3–4 cm^2^, GOA: 3–5 cm^2^) (43–48). Valvular hemodynamics consisted of a high-velocity jet during the ejection phase and intense vortices characterized by strong rotationality near the tip region of the leaflets during the deceleration phase. Diastole was characterized by a reduction in valvular flow momentum, the migration of the tip vortices into the coronary arteries and aorta, and their progressive dissipation. These characteristics are consistent with previous valvular and coronary flow characterizations reported in computational and *in vitro* studies (27, 40, 49, 50). The fWSS captured on the fibrosa was found to be low-magnitude and oscillatory in the base and belly regions, of increasing magnitude from the base to the tip, and dominated by the radial component, as reported previously (40, 41, 51, 52). Lastly, stretch ratios increased from the base to the tip of the leaflets, and the radial stretch component was found to be larger than the circumferential component, as previously reported in the literature (24, 53). This observation is consistent with the leaflet structure and the circumferential alignment of collagen fibers in the fibrosa layer, which contribute to the anisotropy of the leaflet material and its higher stiffness along this direction (54, 55). While the maximum stretch ratios predicted in the present study (radial tS ratio: 1.01–1.10; circumferential tS ratio: 1.01–1.07) are lower than those reported *in vitro* in porcine leaflets (radial: 1.13–1.25; circumferential: 1.09–1.25) (24, 56), they are consistent with those of a previous computational study with an idealized AV geometry (radial: 1.09–1.19; circumferential: 1.09–1.13) (26). Collectively, these observations suggest that the model was able to capture the native normotensive valvular flow and leaflet dynamics.

### 4.2 Effect of hypertension on leaflet mechanics

Our study reveals that elevated aortic pressure altered dramatically AV leaflet function, kinematics and dynamics. The changes predicted affected the leaflet coaptation profile, as well as the leaflet structural and fluid stresses. Our preHTN and HTN models predicted a pressure-dependent decrease in coaptation angle in all leaflets (preHTN: up to 7%; HTN: up to 30%). This result was expected as it reflects the increasing pressure overload imposed by pre-hypertensive and hypertensive aortic flow on the leaflet fibrosa after leaflet closure.

Similarly, the models predicted a pressure-dependent increase in radial and circumferential stretch in all three leaflets. The average increase in stretch ratio captured by the HTN model in the base of the leaflet was +5% in the radial direction and +2% in the circumferential direction relative to normotensive conditions. *in vitro* measurements performed in the same region in porcine leaflets revealed an increase of +8% in the radial direction and +6% in the circumferential direction under an aortic pressure of 160/120 mmHg (24). While the direct comparison of those results reveals substantial differences, it is important to note that the aortic pressure condition implemented in the HTN model was 30 mmHg lower than that used in the *in vitro* study and that substantial variations in stretch ratios were observed experimentally across different valves. Lastly, stretch predictions captured by our models in the base and belly regions indicated only limited differences (<1%) in the LCL and NCL. This observation aligns with *in vitro* results, which revealed no significant difference in stretch between those regions (24).

Alterations in fWSS caused by elevated aortic pressure were complex and found to be pressure magnitude-, leaflet- and component-dependent. Overall, the relatively moderate increase in systolic aortic pressure imposed by the preHTN model (+5 mmHg vs. NTN) was sufficient to subject the leaflets to substantial fWSS overloads (31%–42% increase vs. NTN) in the radial direction (i.e., main flow direction). Interestingly, the additional pressure load imposed by the HTN model during systole (+5 mmHg vs. preHTN) and diastole (+10 mmHg) did not further this effect and resulted in similar fWSS levels. This observation seems to suggest the stronger sensitivity of the fibrosa fWSS environment to changes in systolic aortic pressure than changes in diastolic pressure. A parametric study considering different combinations of systolic and diastolic aortic pressures could provide more insights.

### 4.3 Potential clinical significance

The results collected in the base of the leaflets indicated the maintenance of a low fWSS environment regardless of the pressure condition (TSM < 0.12 Pa), and the complex sensitivity of fWSS magnitude on aortic pressure, with pre-hypertension resulting in sub-physiologic fWSS levels and stage-1 hypertension in supra-physiologic levels. Elevated pressure conditions also increased fWSS bidirectionality in a pressure magnitude-dependent manner in this region on the LCL and NCL. Collectively, those results indicate that preHTN and HTN conditions impose abnormalities in fWSS magnitudes combined with an increasingly bidirectional fWSS environment in the base of some leaflets. Such alterations have been shown to promote signaling pathways similar to those observed in early valvular pathogenesis. Ex vivo studies subjecting porcine AV leaflets to different combinations of physiologic, sub-physiologic and supra-physiologic fWSS magnitude and oscillation in cone-and-plate shear stress devices (57, 58) indicated the maintenance of valvular homeostasis under physiologic fWSS, and the promotion of VCAM1/ICAM1-mediated endothelial activation, BMP4/TGFβ1 paracrine signaling, and MMP/cathepsin catabolic enzyme secretion and activity in the leaflet fibrosa under supra-physiologic fWSS magnitude and oscillatory fWSS directionality (15–19). Another study using a microfluidic device revealed that steady and oscillatory fWSS of similar magnitude (0.2 Pa) to that predicted in the leaflet base under hypertensive conditions (0.1 Pa) promoted endothelial-to-mesenchymal transformation and inflammation when compared with cells exposed to fWSS one-order-of-magnitude higher (59).

The present models also suggested substantial stretch alterations in the base of all three leaflets, marked by a pressure magnitude-dependent increase in radial stretch (up to 3% and 6% increase under preHTN and HTN condition, respectively). Ex vivo studies using a custom stretch bioreactor subjecting porcine AV leaflets to realistic uniaxial stretch (20) indicated that supra-physiologic stretch (15%) upregulated the expression of proteolytic enzymes (MMPs, cathepsins), pro-inflammatory cytokines (BMP2, BMP4) and osteoblastic differentiation markers (Runx2) in the leaflet fibrosa as compared to physiologic stretch (10%) (21, 22).

Altogether, the mechanical abnormalities captured in the leaflet base under pre-hypertensive and hypertensive conditions and the demonstrated ability of similar mechanical alterations to trigger pro-inflammatory and pro-remodeling pathways in AV leaflets suggest the potential existence of a mechano-etiology for CAVD in hypertensive patients, which could also explain the prevalence of this disease in this patient population. However, the thorough demonstration of the implication of hypertensive mechanical stress abnormalities in CAVD progression remains to be established. The detailed description of the leaflet fWSS and tS environments described in this study provides the necessary mechanical prerequisites for future cell and tissue culture studies aimed at elucidating the effects of hypertension on AV biology and testing the mechano-potential etiology of CAVD in hypertensive states.

## 5 Study limitations

### 5.1 Model geometry

The modeling strategy implemented in this study includes several limitations. First, the aortic root geometry consisted of three identical, equi-angularly spaced leaflets and sinuses. This is a simplification of the native anatomy, which includes non-identical leaflet and sinus sizes and shapes (29). Therefore, the functional and hemodynamic differences captured between different leaflets in the present study are expected to be exacerbated in the native valve.

### 5.2 Material formulation

The Mooney-Rivlin formulation used to model the leaflet material is only an approximation of the biological material characteristics which include anisotropy, viscoelasticity and a tri-layered structure. Anisotropy results from the different orientations of collagen and elastin fibers throughout the leaflet layers. While the isotropic material formulation considered in our model discards the differences in tissue mechanical behaviors in the radial and circumferential directions, this formulation has been adopted in prior FSI valve models (26, 36, 41) and has been shown to generate only limited differences in leaflet mechanics relative to models implementing an anisotropic leaflet material (60, 61). Therefore, while the anisotropy of the leaflet material could potentially alter the leaflet curvature during valve closure and the circumferential tensile stretch predictions, its impact on the regional leaflet fluid wall shear stress is expected to be weak.

Although the isotropic approximation can capture global hemodynamics and leaflet kinematics with reasonable accuracy, the simplified material formulation resulted in a non-physiologic valvular resistance to flow. As a result, the physiologic cardiac output of 5 L/min was achieved while subjecting the valve to a non-physiologic transvalvular pressure gradient. However, since all three models (NTN, preHTN and HTN) implemented a similar approach, the hemodynamic differences captured between the models are meaningful and provide reliable insights into how aortic pressure may impact valvular function.

### 5.3 Flow regime

Peak-systolic flow through the valve is known to become in transition to the turbulent flow regime. However, the present study implemented a laminar flow model. Although the investigation of the native valvular hemodynamics would clearly benefit from an approach capturing the features of this transitional flow, turbulence modeling approaches remain difficult to implement in the context of a fully coupled FSI problem. Reynolds-averaged Navier Stokes (RANS)-based turbulence models have been primarily developed and tuned for fully developed turbulence, which are not suitable for transitional and relaminarizing flows such as that through the valve. Such models are also typically too dissipative and their use to simulate valvular flow would lead to incorrect hemodynamic predictions. Large-eddy simulation (LES) models are also available and offer a good compromise between the RANS approach and direct numerical simulations (DNS). However, they still require high mesh densities and small time steps, which complicates their use for valvular FSI simulations. Therefore, while modeling blood flow as laminar may not capture the brief transition to turbulence occurring at peak systole, the laminar assumption permits to capture the near-native hemodynamic characteristics in the aortic root during most of the cardiac cycle, and is consistent with prior FSI valve models (26, 41).

### 5.4 Boundary conditions

A uniform pressure load of 80 mmHg (10.7 kPa) was applied on the leaflet fibrosa to ensure proper leaflet coaptation during diastole. While this condition is not equivalent to the pressure load that would result from the prescription of a pressure at the outlet of the fluid domain, this modeling approach has been implemented and validated in other computational valve studies to characterize the leaflet structural deformations in the absence of flow (62–64).

In addition, the implementation of the ALE method required to maintain a single fluid domain throughout the cardiac cycle. This was achieved by using a small contact offset in the structural domain, which prevented the leaflets from completely closing and splitting the fluid domain during leaflet coaptation. In doing so, a small orifice was still present even after closure, causing a small residual orifice and leakage during early diastole. As a consequence, early-diastolic flow structures should be interpreted with caution. While other modeling approaches such as Contact Zones and Contact Marks methods available in ANSYS Fluent could prevent leakage by applying porous resistance or a zero-mass-flux boundary, respectively, these approaches impose solver and discretization constraints and can require additional stabilization and time-step reduction in transient runs.

Lastly, the implementation of three-element Windkessel (RCR) models at the aortic and coronary outlets could enhance the accuracy of the simulations by capturing the resistive and compliant properties of the vascular system. However, this approach could not be implemented in the present study due to the requirement to maintain complete control over the aortic pressure to enforce different hypertensive levels. In addition, the same coronary flow rates were imposed in all three models to capture the long-term changes in coronary perfusion pressure and coronary resistance triggered by sustained hypertensive conditions, and account for the well-established autoregulatory capacity of the coronary circulation, which results in the maintenance of a relatively constant myocardial perfusion across a wide range of coronary perfusion pressure (38). The use of a RCR model at the coronary outlets would not guarantee the maintenance of the same coronary flow rate under normotensive and hypertensive conditions. Nevertheless, the absence of RCR coupling likely contributed to artifacts in diastolic hemodynamics (e.g., residual forward jet) and prevented the direct reproduction of physiological waveforms.

## 6 Conclusion

The present study provides new insights into the impact of systemic hypertension on AV function and compelling support for the existence of a mechano-etiology of CAVD in hypertensive patients. Further experimental validation using flow diagnosis and cell/tissue culture techniques is needed to confirm the mechanical stress alterations predicted under hypertensive states and to ascertain their potential role in CAVD development. Should causality between hypertensive hemodynamics and valvular pathogenesis be demonstrated, different diagnosis and treatment strategies could be designed to better identify the patients at risk, and to deactivate or attenuate the mechano-sensitive pathways contributing to CAVD progression.

## Data Availability

The raw data supporting the conclusions of this article will be made available by the authors, without undue reservation.

## Supplementary material

Supplementary Video 1 MP4 Animation of velocity vector and vorticity contour fields in the RCL- LCL- and NCL-bisecting planes for the NTN, preHTN and HTN cases over one cardiac cycle. Playback speed: 0.25×. White ball indicates the current phase of the cardiac cycle on the cardiac output waveform.

Supplementary Video 2 MP4 Animation of the fWSS contour field on the fibrosa of the RCL, LCL and NCL for the NTN, preHTN and HTN cases over one cardiac cycle. Playback speed: 0.25×. White moving ball indicates the current phase of the cardiac cycle on the cardiac output waveform.
